# Depression amongst patients commencing maintenance dialysis is associated with increased risk of death and severe infections: A nationwide cohort study

**DOI:** 10.1371/journal.pone.0218335

**Published:** 2019-06-13

**Authors:** Ping-Hsun Wu, Ming-Yen Lin, Teng-Hui Huang, Yi-Ting Lin, Chi-Chih Hung, Yi-Chun Yeh, Hung-Tien Kuo, Yi-Wen Chiu, Shang-Jyh Hwang, Jer-Chia Tsai, Juan-Jesus Carrero

**Affiliations:** 1 Division of Nephrology, Department of Internal Medicine, Kaohsiung Medical University Hospital, Kaohsiung Medical University, Kaohsiung, Taiwan; 2 Institute of Clinical Medicine, College of Medicine, Kaohsiung Medical University, Kaohsiung, Taiwan; 3 Faculty of Renal Care, College of Medicine, Kaohsiung Medical University, Kaohsiung, Taiwan; 4 Master of Public Health Degree Program, College of Public Health, National Taiwan University, Taipei, Taiwan; 5 Department of Family Medicine, Kaohsiung Medical University Hospital, Kaohsiung Medical University, Kaohsiung, Taiwan; 6 Department of Family Medicine, Kaohsiung Municipal Hsiao–Kang Hospital, Kaohsiung, Taiwan; 7 Department of Psychiatry, Kaohsiung Medical University Hospital, Kaohsiung Medical University, Kaohsiung, Taiwan; 8 Department of Psychiatry, College of Medicine, Kaohsiung Medical University, Kaohsiung, Taiwan; 9 Department of Medical Epidemiology and Biostatistics (MEB), Karolinska Institutet, Stockholm, Sweden; University of Mississippi Medical Center, UNITED STATES

## Abstract

**Background:**

Depression is common in dialysis patients, but the clinical impact of this condition is poorly defined.

**Methods:**

Out of 57,703 patients starting dialysis during 2000–2007 recorded in the National Health Insurance Research Database of Taiwan, we identified 2,475 patients with a clinical diagnosis of depression, and compared them with 1:5 age- and sex-matched patients without a depression diagnosis (n = 12,375). Patients were followed up for hospitalisation due to severe infections, major adverse cardiovascular events (MACE) and death. Multivariable Cox regression and competing risk analyses (accounting for death when appropriate) were used to estimate risk associations.

**Results:**

Patients with depression had a higher frequency of comorbidities. During a mean follow-up of 3.2 years, 1,140 severe infections, 806 MACE, and 1,121 deaths were recorded. Compared to controls, patients with depression were at increased risk of death (adjusted hazard ratio 1.24; 95%CI 1.16–1.33). Patients with depression were also at higher risk of severe (1.14; 1.06–1.22) and fatal infections (death within 30 days, 1.22; 1.09–1.35), attributed mainly to sepsis (1.19; 1.08–1.31), septic shock (1.36; 1.13–1.62) and pneumonia (1.19; 1.07–1.33). Conversely, no association was observed between depression and the MACE risk (1.04; 0.94–1.15).

**Conclusion:**

Dialysis patients with depression are associated with increased risk of infections and death.

## Introduction

Depression is increasingly common in modern society and projected to be the future major cause of disability worldwide [1]. Depression considerably reduces the quality of life and frequently accompanies a number of chronic diseases [2], including chronic kidney disease (CKD). Indeed, between 20 to 30% of patients with severe CKD suffer from depression [3–6]. Even if lacking a formal diagnosis of depression, patients with CKD were often reported as having the heavy burden of depression symptoms [7] and depression is considered a priority research subject among patients on or nearing dialysis [8].

In the wider community, growing evidence links depression with the risk of death [9], cardiovascular [10–13] and infectious [14, 15] complications, attributed to both plausible biological (e.g. inflammation-mediated) and psychosocial mechanisms (e.g. attitude towards disease). Depression has been consistently associated with increased mortality risk among patients receiving dialysis [3], but whether depression is associated with cardiovascular/infection events in these patients has not been well explored. Previous studies show inconsistencies in the association of depression with adverse outcomes [5, 16–20], often hampered by small sample sizes, outcome ascertainment bias, inadequate adjustment for confounding variables and short-term follow-up.

Given the exacerbated cardiovascular and infection risk to patients undergoing dialysis [21, 22], characterising these associations is relevant to inform patients and healthcare policymakers about appropriate prevention strategies and health service planning. Depression in patients with end-stage renal disease (ESRD) may lead to adverse outcomes, a care burden for the family, and a higher cost to society. It is worthwhile investigating the risks of adverse outcomes and their impacts caused by depression in ESRD patients. We decided to evaluate the possible impact of depression on clinical outcomes among incident dialysis patients using the National Health Insurance Research Database (NHIRD) in Taiwan. Outcomes addressed included death, major adverse cardiovascular events (MACE) and severe infections.

## Materials and methods

### Data source

In 1995, Taiwan launched a compulsory social insurance program, National Health Insurance (NHI), to provide healthcare for all residents. Coupled with NHI, the NHIRD (National Health Insurance Database) was initiated, gathering detailed healthcare data for >99% of Taiwan’s population. NHIRD contains a registry system for "Catastrophic Illnesses". Insured population with severe diseases, including end-stage renal disease, are eligible for registration with a catastrophic illness to remit the co-payment. The database includes all relevant healthcare information, including diagnostic codes (in the format of the *International Classification of Disease*, *Ninth Revision* [*ICD-9*]), date of diagnosis, date of death, drug prescriptions, and outpatient/ inpatient claims. The inclusion in the dialysis register requires the medical examination of two nephrologists who investigate the underlying disease, laboratory data, renal ultrasonography, and indications for dialysis treatment. For the protection of privacy, patients were de-identified before analysis; therefore, informed consent was waived because of the use of anonymised data. This retrospective observational study complied with the Declaration of Helsinki guidelines and was approved by the research ethics board of Kaohsiung Medical University Hospital (KMUH-IRB-EXEMPT-20140047).

### Study cohort

**Fig 1:**
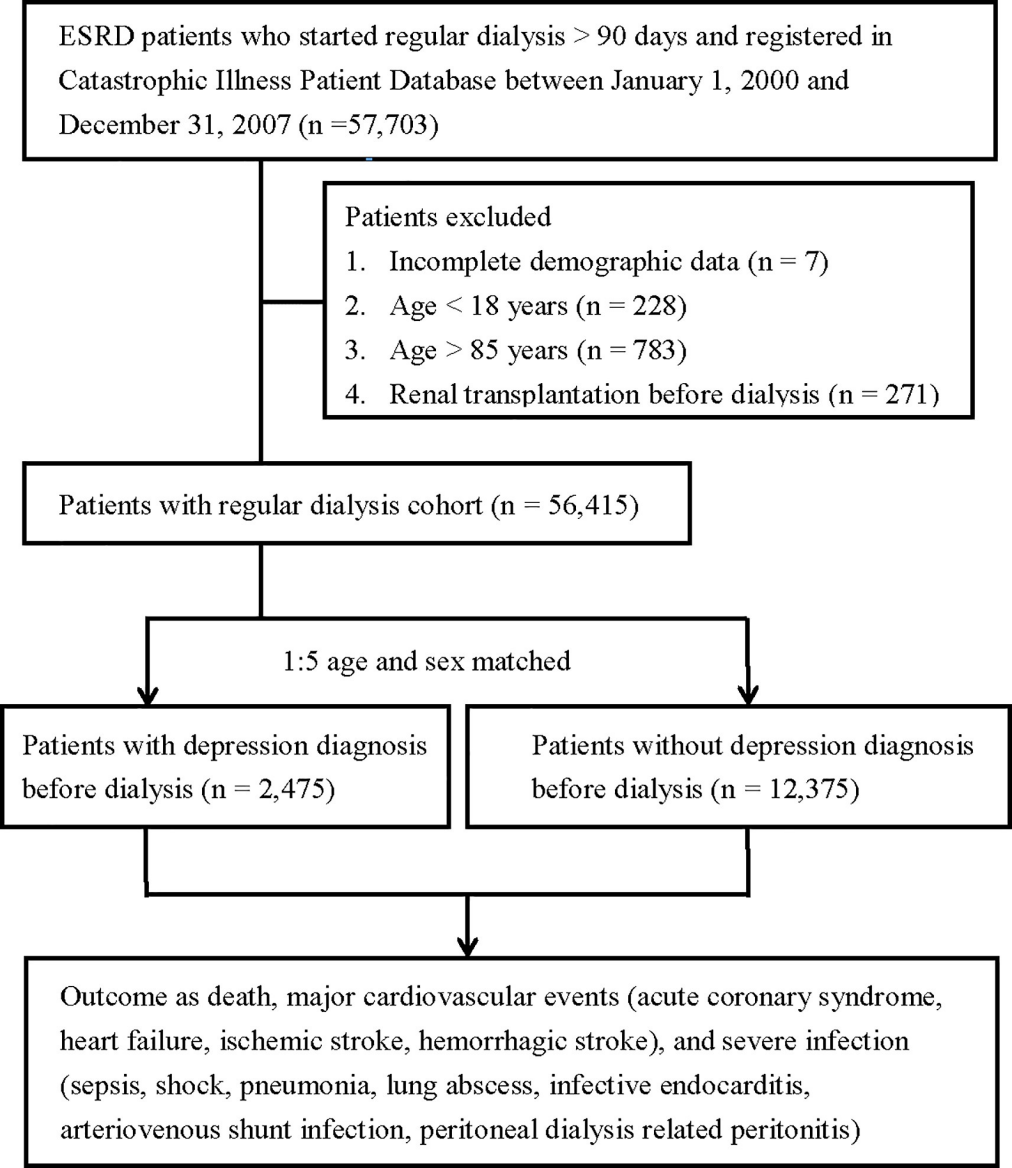
Patient selection flow chart.

We enrolled all patients initiating chronic dialysis for more than 90 days (see S1 Table for all study definitions), either HD or PD between January 1, 2000, and December 31, 2007 (n = 57,703) (Fig 1). After excluding patients with incomplete demographic data (n = 7), under 18 years of age (n = 228) and over 85 years of age (n = 783), and those receiving renal transplantation before dialysis (n = 271), the remaining 56,414 patients were eligible for our study.

### Study exposure

The study exposure was clinically diagnosed depression, defined as the presence of a depression *ICD-9* diagnostic code in at least two outpatient claims within 12 months prior to the index date (initial dialysis) or with once depression diagnostic codes in an inpatient claim (n = 2,475). The ICD-9 codes used for depression were 296.2 for major depressive disorder and a single episode, 296.3 for major depressive disorder or a recurrent episode, 300.4 for dysthymic disorder, and 311 for depressive disorder or not elsewhere classified. These ICD-9 codes used were based on the Diagnostic and Statistical Manual of Mental Disorder (DSM) depression diagnosis codes (296.2, 296.3, 300.4, or 311) and had been used previously [23–25]. For each patient with depression, we randomly selected from the total dialysis cohort five control patients matched by age and sex (n = 12,375).

### Study outcomes and follow-up ascertainment

The *study outcomes* were (all-cause) mortality, major adverse cardiovascular events (MACE), and severe infections. Deaths data was collected from the Catastrophic Illness Database. MACE included the composite of ACS, heart failure, ischemic stroke, and haemorrhagic stroke. The diagnosis has been validated in previous studies [26, 27]. Severe infection entailed hospitalisation admissions due to sepsis, pneumonia, lung abscess, infective endocarditis, arteriovenous shunt infection, or peritoneal dialysis-related peritonitis, whichever occurred first (S1 Table).

In addition, we also evaluated the risk of fatal infections, defined as an infection related hospitalisation followed by death within 30 days (S1 Table) and explored single cardiovascular and infections events to explore result consistency.

### Study covariates

Comorbid history (diabetes mellitus [DM], hypertension, hyperlipidaemia, coronary artery disease, cerebrovascular disease, autoimmune disease, malignancy, alcohol dependence, psychotic disorders, anxiety disorders, and sleep disorders) was identified at baseline by the presence of ICD-9 codes (listed in S1 Table) in at least two outpatient claims (>90 days apart to avoid accidental inclusion of miscoded patients) or in at least one inpatient claim during the year before the start date of dialysis [28]. We also reconstructed information on concurrent medication, including antiplatelets/warfarin, anti-hypertensive drugs, statins, oral antidiabetic agents, insulin, antipsychotic agents, benzodiazepines and hypnotics, as identified by Anatomical Therapeutic Chemical (ATC) codes (S2 Table). Urbanisation levels were divided into two strata and economic status was classified into three categories: low, moderate, and high income; less than 20,000 New Taiwan dollars (NTD) monthly; or ≥20,000 NTD monthly (US $1 = NTD 32.1 in 2008).

### Statistical analyses

The distribution of demographic data and comorbidities between the depression and control groups was described using mean ± standard deviation for continuous variables and count (percentage) for categorical variables, and compared using chi-square test and independent t test. Patients were followed from index date to event, loss to follow-up, or until the end of 2008. Cumulative event incidences were estimated using the Fine and Grey method and log-rank test. Subdistribution hazard models were used for comparing the risks of MACE and infection accounting for death by other causes as the competing risk [29]. After ensuring the fulfillment of proportional hazards assumption by Schoenfeld residuals trend tests, we applied either traditional Cox models or subdistribution hazards models to estimate unadjusted and adjusted hazard ratios (HRs) and 95% confidence intervals (CIs) [30]. To evaluate the consistency of our findings, we carried out a series of stratification analyses in the following populations: age categories, men/women, haemodialysis/peritoneal dialysis, and presence/absence of DM, hypertension, hyperlipidaemia, coronary artery disease, cerebrovascular disease, autoimmune disease, and malignancy. Analyses utilised SAS statistical software (version 9.3; SAS Institute Inc.). A two-tailed *p* value less than 0.05 was considered statistically significant.

To assess the robustness of our findings, we performed a series of sensitivity analyses, including: (1) redefining depression diagnosis by only the major depression diagnostic codes (ICD-9 codes 296.2 and 296.3); (2) redefining depression diagnosis as the presence of any depression diagnostic code in at least two outpatient claims or one inpatient claim and the use of any depression-related medications (S2 Table); (3) redefining depression diagnosis as the presence of any depression diagnostic code in at least two outpatient claims or one inpatient claim and regular follow-up at outpatient psychiatric clinic; (4) using a logistic regression model that included age, sex, comorbidities, and concomitant medication use as covariates to compute the propensity score and performing analysis based on patients matched by their propensity to have depression.

## Results

The distribution of socio-demographic characteristics, comorbid medical disorders, and medications used by patients with depression and controls are shown in Table 1. Compared to controls, patients with depression had a slightly lower socioeconomic status, and presented with a higher frequency of comorbidities. They also more frequently used antipsychotic agents, benzodiazepines, and hypnotics.

**Table 1:** Baseline characteristics among dialysis patients with and without depression.

	Depression	Controls	
	(n = 2,475)	(n = 12,375)	
Characteristic	N (%)	N (%)	*p* value
**Age, years (mean ± SD)**	62.6 ± 12.3	62.6 ± 12.3	0.9
**Age group**			>0.99
18–39 years	109 (4.4%)	545 (4.4%)	
40–59 years	870 (35.2%)	4,350 (35.2%)	
60–85 years	1,496 (60.4%)	7,480 (60.4%)	
**Sex**			>0.99
Men	901 (36.4%)	4,505 (36.4%)	
Women	1,574 (63.6%)	7,870 (63.6%)	
**Dialysis modality**			0. 4
Haemodialysis	2,326 (94.0%)	11,570 (93.5%)	
Peritoneal dialysis	149 (6.0%)	805 (6.5%)	
**Urbanization level**			0.7
City area	1,773 (71.7%)	8,824 (71.4%)	
Rural area	699 (28.3%)	3,531 (28.6%)	
**Socioeconomic status**			0.02
Low	944 (38.1%)	4,638 (37.5%)	
Moderate	1,088 (44.0%)	5,226 (42.2%)	
High	443 (17.9%)	2,511 (20.3%)	
**Comorbidities**			
Diabetes mellitus	1,355 (54.7%)	6,343 (51.3%)	0.002
Hypertension	2,086 (84.3%)	10,082 (81.5%)	0.001
Hyperlipidaemia	587 (23.7%)	2,504 (20.2%)	<0.001
Coronary artery disease	747 (30.2%)	3,077 (24.9%)	<0.001
Heart failure	660 (26.7%)	3,298 (26.7%)	>0.99
Cerebrovascular disease	548 (22.1%)	1,481 (12.0%)	<0.001
Autoimmune disease	61 (2.5%)	231 (1.9%)	0.06
Malignancy	207 (8.4%)	845 (6.8%)	0.007
Alcohol dependence	23 (0.9%)	86 (0.7%)	0.3
Psychotic disorder	76 (3.1%)	62 (0.5%)	<0.001
Anxiety disorder	375 (15.2%)	407 (3.3%)	<0.001
Sleep disorder	660 (26.7%)	1,214 (9.8%)	<0.001
**Medications use**			
Antiplatelets/Warfarin	1,143 (46.2%)	5,697 (46.0%)	0.9
Anti-hypertensive drugs	1,880 (76.0%)	9,224 (74.5%)	0.1
Statins	757 (30.6%)	3,735 (30.2%)	0.7
Oral antidiabetic agents	791 (32.0%)	3,739 (30.2%)	0.09
Insulin	667 (26.9%)	2,967 (24.0%)	0.002
Antipsychotic agents	761 (30.7%)	1,798 (14.5%)	<0.001
Benzodiazepines	1,480 (59.8%)	5,399 (43.6%)	<0.001
Hypnotics	1,506 (60.8%)	4,935 (39.9%)	<0.001
**Year of dialysis start**			<0.001
2000–2003	1,025 (41.4%)	5,667 (45.8%)	
2004–2007	1,450 (58.6%)	6,708 (54.2%)	
**Follow-up years**			
Mean ± SD	3.2 ± 2.1	3.6 ± 2.2	<0.001
Median (IQR)	2.7 (1.5–4.6)	3.1 (1.7–5.1)	<0.001

**Fig 2:**
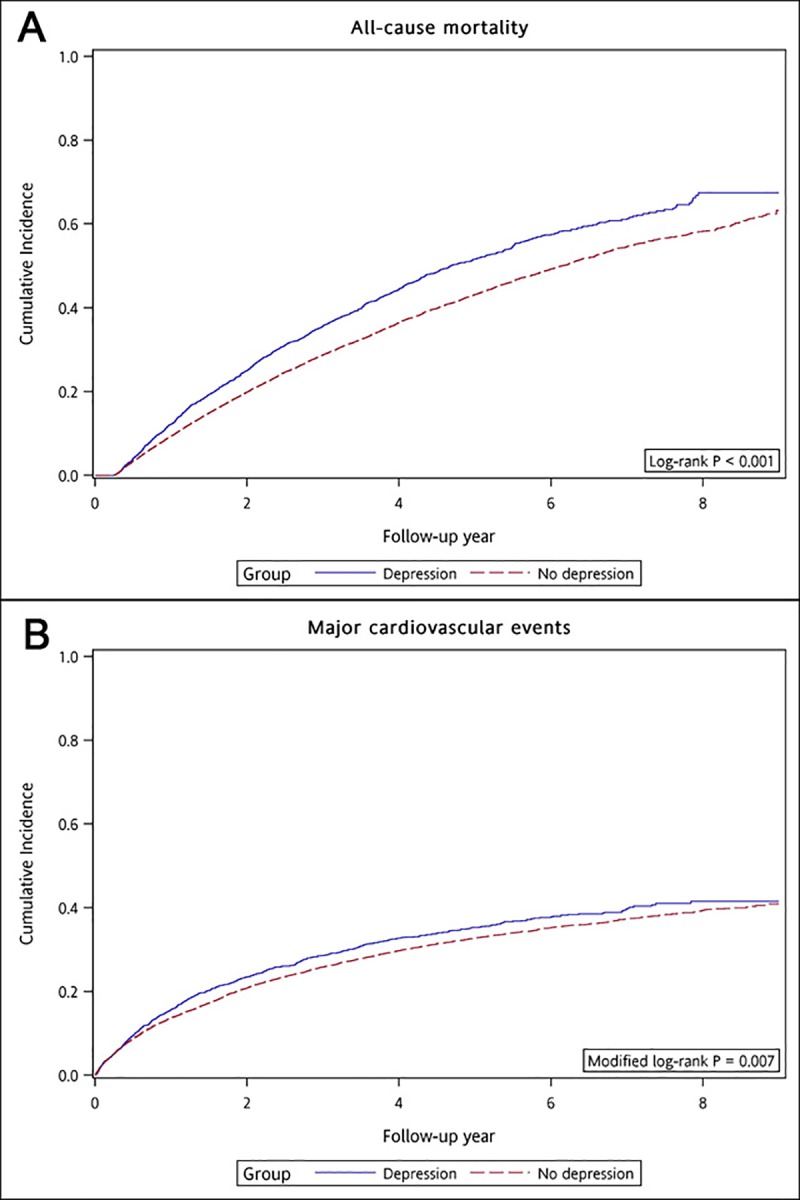
Cumulative incidence of (A) death and (B) major cardiovascular events in incident dialysis patients with vs without depression. For the cumulative incidence of major cardiovascular events, Fine-Gray methods were performed accounting for the competing risk of death.

During a mean follow-up of 3.2 years, we observed a higher crude cumulative incidence of death in patients with depression compared to controls (45.29% versus 39.52%; Log-rank *p* <0.001) (Fig 2A and S3 Table). Multivariable Cox proportional hazards regression analyses consequently showed that compared to controls, dialysis patients with depression were at higher risk of death (final model adjusted HR: 1.24 [95% CI: 1.16–1.33]; *p* <0.001) (Table 2).

**Table 2:** Clinical outcomes associated with depression among incident dialysis patients.

Variable	Overall events (Depression/Control)		Adjusted Hazard Ratio (95% CI)	
	Event numbers	Incidence rate^#^	Model 1^†^	Model 2^‡^
**Death**^§^	1,121 / 4,890	142.22 / 111.28	1.15 (1.07–1.23)***	1.24 (1.16–1.33)***
**MACE**	806 / 3,787	94.07 / 81.73	1.03 (0.95–1.12)	1.03 (0.95–1.12)
**Severe infections**	1,140 / 4,990	152.06 / 117.04	1.16 (1.08–1.24)***	1.14 (1.06–1.22)***
**Fatal infections**	513 / 2,100	52.06 / 39.4	1.19 (1.08–1.32)***	1.22 (1.09–1.35)***

MACE defined as the composite of acute coronary syndrome, heart failure, ischemic stroke, and hemorrhagic stroke.

^†^Model 1: Adjusted for comorbid disorders (diabetes mellitus, hypertension, hyperlipidaemia, coronary artery disease, cerebrovascular disease, autoimmune disease, malignancy, alcohol dependence, psychotic disorder, anxiety disorder, sleep disorder), and competing risk of death (when appropriate).

^‡^Model 2: Adjusted for comorbid disorders, medications (antiplatelets/warfarin, anti-hypertensive drugs, statins, oral antidiabetic agents, insulin, antipsychotic agents, benzodiazepines, hypnotics), and competing risk of mortality

^§^ Analyses predicting death and fatal infections used Cox-proportional hazard models; Analyses predicting MACE or severe infections used Fine and Gray models accounting for the competing risk of death.

^#^Incident rate (per 1000 person-years)

*p<0.05

**p<0.01

***p<0.001

**Fig 3:**
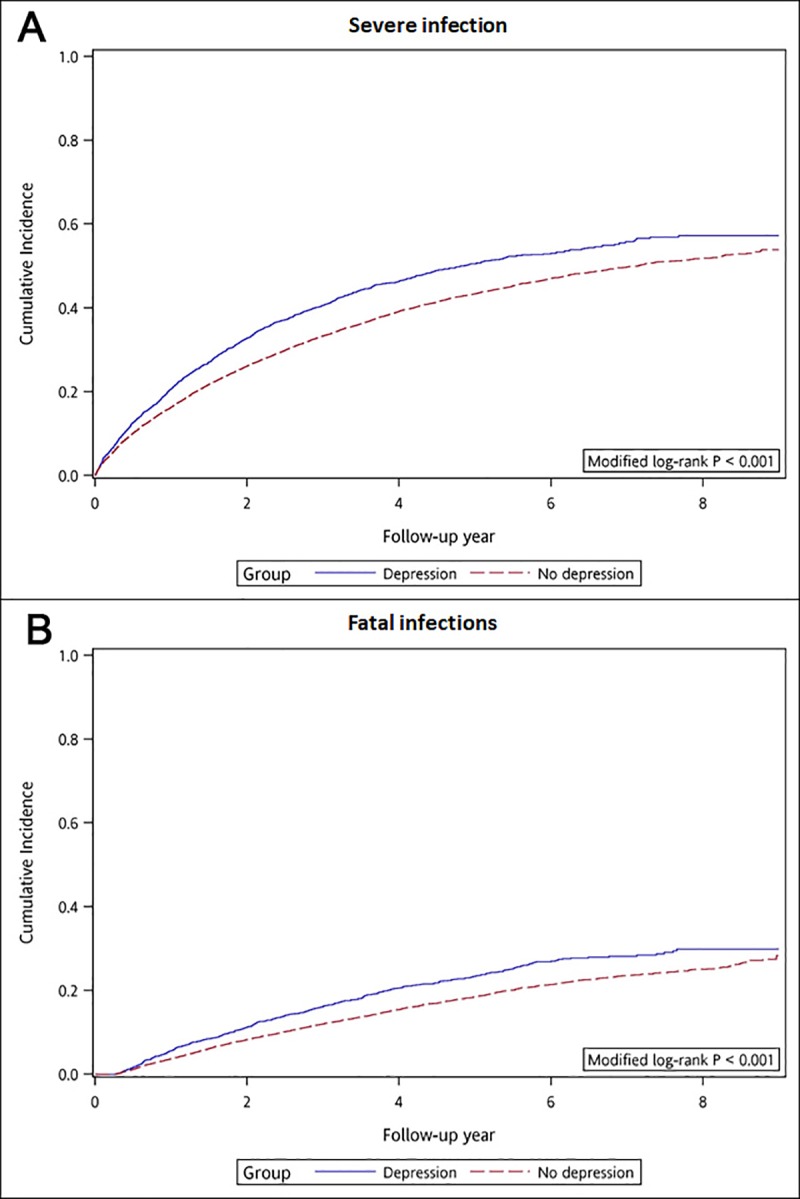
Cumulative incidence of (A) severe infections and (B) fatal infections in incident dialysis patients with vs without depression. For the cumulative incidences of severe infections, Fine-Gray methods were performed accounting for the competing risk of death.

**Fig 4:**
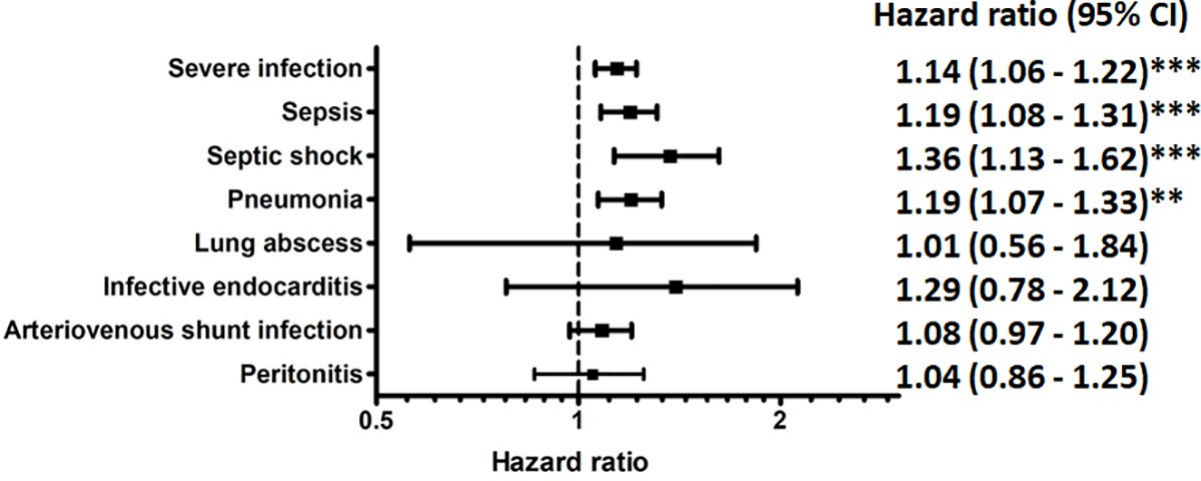
Risks of single infection events associated with clinical depression. Footnote: Subdistribution hazard Model: Adjusted for comorbid disorders (diabetes mellitus, hypertension, hyperlipidaemia, coronary artery disease, cerebrovascular disease, autoimmune disease, malignancy, alcohol dependence, psychotic disorder, anxiety disorder, sleep disorder), medications (antiplatelets/warfarin, anti-hypertensive drugs, statins, oral antidiabetic agents, insulin, antipsychotic agents, benzodiazepines, hypnotics), and competing risk of mortality *p<0.05, **p<0.01, ***p<0.001.

Patients with depression had a higher crude cumulative incidence of (fatal) infections than controls (46.06% versus 40.32% for severe infections; Log-rank *p*<0.001 and 20.73% versus 16.97% for fatal infections; Log-rank *p*<0.001) (Fig 3 and S3 Table). Multivariable Cox regression analyses adjusting for comorbidities and concomitant medications showed that depression was associated with a 14% higher risk of severe infections (adjusted HR: 1.14 [95% CI: 1.06–1.22]; *p*<0.001) and a 22% higher risk of fatal infections (adjusted HR: 1.22 [95% CI: 1.09–1.35]; *p*<0.001) (Table 2). When analyzing single infection types, patients with depression were associated with a higher risk of sepsis (adjusted HR: 1.19 [95% CI: 1.08–1.31]; *p*<0.001), septic shock (adjusted HR: 1.36 [95% CI: 1.13–1.62]; *p*<0.001), and pneumonia (adjusted HR: 1.19 [95% CI: 1.07–1.33]; *p*<0.001). Other studies of infection types did not seem to differ significantly between the study groups (Fig 4).

Conversely, the rate of MACE was higher in patients with depression than controls (Fig 2B). However, Multivariable Cox regression did not report differences between both groups (Table 2, adjusted HR for MACE: 1.03 [95% CI: 0.95–1.12; *p* = 0.5]). Single cardiovascular events did not associate with the exposure of depression (S4 Table).

The association between depression, death and infections were consistent throughout a variety of patient subpopulations S1–S3 Figs). Likewise, the lack of association between depression and MACE was also consistent (S2 Fig). Identifying cases with depression by alternative algorithms (Approaches 1–3 in S5 Table) yielded similar results, as well as after performing a propensity score-matched analysis (Approach 4 in S5 Table).

## Discussion

In this large nationwide cohort of incident dialysis patients, we show that clinically diagnosed depression was associated with the risk of infectious complications and death. Conversely, there was no apparent association between depression and MACE.

Infection-related hospitalizations contribute substantially to excess morbidity and mortality in patients undergoing chronic dialysis, and infection is the second leading cause of death in this population [31]. A novel and clinically relevant finding in our study is the consistent association between depression and the risk of severe (fatal) infections. Our finding is backed up by a recent observational report of 2200 prevalent HD patients undertaking the Beck Depression Inventory (BDI) II questionnaire and showing that non-cardiovascular causes mainly accounted for the association between depression and death [19]. Although the authors reported infections as one of the main non-CVD causes of death, the study lacked statistical power to assess the risk of infection-related mortality. Our careful analysis of causes of infection suggested the association to be mainly accounted for by a higher risk of sepsis and pneumonia. Previous studies have consistently established the relationship between depression and pneumonia in the general population [14, 15], and we hereby expand that finding to dialysis populations. This is important, given that pneumonia is one of the most common causes of hospitalisation for these patients [31]. It has been suggested that peritoneal dialysis patients with depression are at higher risk of peritonitis [18], a finding that could not be observed in our study. However, haemodialysis is the primary mode of long-term dialysis in Taiwan, estimated at 90%. We acknowledge that this study may not be strong enough to establish these associations.

Mechanisms to explain the association between depression and infection risk are not fully elucidated, but likely involve both biological and psychosocial pathways. Depression may arise as a consequence of the high comorbidity burden of these patients and the lifestyle changes that chronic dialysis imposes in them. Supporting this notion, Bolware *et al*. [32] observed attenuated cardiovascular risks from time-lag analyses among 917 dialysis patients with repeated depression symptom assessments, which may indicate a partial role for reverse causality or that medical comorbidity may precede depressive symptoms. Although residual confounding is always present in observational studies such as ours, adjustment for the most relevant comorbidity domains in these patients did not abrogate the association. Further, depression can also lead to lower treatment adherence [33, 34] and lower compliance with several aspects of disease management such as adherence to fluid restrictions [35] that may explain this risk. In addition, previous studies in the general population have demonstrated that patients with depression have exhibited more severe and more prolonged inflammatory responses after antigen challenge, suggesting that depression may result in immune dysregulation [36, 37]. Depression and psychological stress can result in increased proinflammatory cytokine release [38–40], decreased lymphocyte function and immune cell activity [41]. Bereavement has also been shown to decrease the production of neutrophil superoxides [42] and to reduce the functional activity of natural killer cells [43]. These mechanisms have been collectively implicated in the impaired wound healing and increased risk of infection reported among individuals with depression and other affective mood disorders [44, 45].

Our observed association between depression and the risk of mortality agrees with previous studies recently meta-analyzed [3] and altogether these are likely to bring attention to a patient subpopulation at high risk of adverse outcomes. In view of the associations between depression and CVD events in non-CKD studies [10–13] and of the association between depression with CVD risk factors such as inflammation and malnutrition in dialysis patients [46], it has been hypothesized that these patients may be at a heightened CVD risk. We did not observe any such association in our study. Although a lack of power to detect this association is possible, we note that previous reports do not seem to observe a relationship between depression and CVD risk either [19, 32].

As for clinical implications, our study supports the need for collaborative care interventions integrating mental health within the regular medical care of dialysis patients. Such collaborative programs could reduce depressive symptoms, improve functioning [47, 48] and improve chronic medical illness management [48]. Furthermore, our results may inform healthcare policymakers and patients about the need for appropriate prevention strategies. If our results are confirmed in other settings, it may be plausible to consider patients with depression as eligible for vaccination campaigns, for instance. Ventilators, central venous catheters, and urinary catheters, frequently used in these patients, are significant sources of infection. Thus, it may also be critical to handle these medical devices with infection-preventive measures.

This study should be viewed in light of several strengths and limitations. Strengths are its national representativeness with complete ascertainment of comorbidities and outcomes given that NHI is a compulsory and universal healthcare system. Further, we employed validated algorithms to identify cases, comorbidities and outcomes. Limitations include the observational nature of our findings, which preclude any conclusion on causality or directionality of the associations. We also acknowledge that we could only select individuals with clinically diagnosed depression, possibly resulting in misclassification bias. Depression is still subjected to social stigma, and some patients may have difficulty acknowledging their symptoms or seeking care. Since the identification of depressive patients was based on physician diagnosis, the prevalence of depressive disorders is probably underestimated. This limitation would, however, have resulted in an underestimation of the observed associations. Selection bias may also occur, as sicker patients accessing the health system more frequently may well be more likely to have depression recognized and recorded–and will be more likely to die over the follow-up period. Our attempts to mitigate this included a rich multivariable adjustment for comorbidities, medications and treatment characteristics. We acknowledge that some confounding factors such as alcohol consumption, dietary habits, physical activity, family history of depression, and haemodynamic stability during dialysis sessions were not available. Furthermore, death was obtained in the catastrophic illness dataset. However, the encrypted NHIRD was not linked to the national death registry, so the ascertainment of specific causes of death is limited in our study. Thus, this result should be cautiously interpreted. Finally, the study was conducted in patients of Han Chinese ethnicity, and extrapolation of these findings to other settings should be done with caution.

### Conclusion

We conclude that incident dialysis patients with depression are at increased risk of infection and death. Sepsis, septic shock, and pneumonia were the infection types most markedly linked to depression. Increasing patient and health care provider awareness of this differential pattern of risk could have benefits for patient management, prevention strategies, and health service planning.

## Supporting information

S1 Checklist STROBE statement—Checklist of items that should be included in reports of cohort studies. (DOCX)

S1 Table ICD-9-CM codes used to identify clinical conditions. (DOCX)

S2 Table Anatomical Therapeutic Chemical (ATC) code used to define medication use. (DOCX)

S3 Table Incidence and incidence rate ratio of study outcomes among incident dialysis patients. (DOCX)

S4 Table Risks of single cardiovascular events associated to depression. (DOCX)

S5 Table Sensitivity analyses using alternative algorithms to identify cases with depression. (DOCX)

S1 Fig Stratified analysis for death associated with depression. (DOCX)

S2 Fig Stratified analysis for major cardiovascular events associated with depression. (DOCX)

S3 Fig Stratified analysis for severe infections associated with depression. (DOCX)

S4 Fig Stratified analysis for fatal infections associated with depression. (DOCX)
