# Supplementary material for: Depression amongst patients commencing maintenance dialysis is associated with increased risk of death and severe infections: A nationwide cohort study
Source: PLoS One. 2019 Jun 13;14(6):e0218335. doi: 10.1371/journal.pone.0218335 (PMC6564035; doi:10.1371/journal.pone.0218335)
Supplement: S1 Table — (DOCX) [file pone.0218335.s002.docx]

**S1 Table. ICD-9-CM codes used to identify clinical conditions**

| Disease | Definition | Definition validated or reported previously |
| --- | --- | --- |
| Hemodialysis | ICD-9 codes 585 with catastrophic illness registration plus procedure code 58001C, 58019C, 58020C, 58021C, 58022C, 58023C, 58024C, 58025C, 58026C, 58027C, 58029C for more than 3 months | Definition reported[1, 2] |
| Peritoneal dialysis | ICD-9 codes 585 with catastrophic illness registration plus peritoneal dialysis-related treatment for more than 3 months | Definition reported[1, 2] |
| Renal transplantation | ICD-9 codes V42.0, 996.81 with catastrophic illness registration | Definition reported[3] |
| Depression | ICD-9 codes 296.2, 296.3, 300.4, 311 | Definition reported[4] |
| Acute coronary syndrome | ICD-9 codes 410, 411 | Definition validation[5, 6] |
| Heart failure | ICD-9 codes 401.91, 402.01, 402.11, 404.01, 404.03, 404.11, 404.91, 404.93, and 428 | Definition reported[7] |
| Ischemic stroke | ICD-9 codes 433, 434, 436 | Definition validation[8, 9] |
| Hemorrhagic stroke | ICD-9 codes 430, 431, 432 | Definition reported[10, 11] |
| Sepsis | ICD-9 codes used to identify bacterial/fungal infections (001.x, 002.x, 003.x, 004.x, 005.x,008.x, 009.x, 010.x, 011.x, 012.x, 013.x, 014.x, 015.x, 016.x, 017.x, 018.x, 137.x, 020.x, 021.x, 022.x, 023.x, 024.x, 025.x, 026.x, 027.x, 030.x, 031.x, 032.x, 033.x, 034.x, 035.x, 036.x, 037.x, 038.x, 039.x, 040.x, 041.x, 090.x, 091.x, 092.x, 093.x, 094.x, 095.x, 096.x, 097.x, 098.x, 100.x, 101.x, 102.x, 103.x, 104.x, 110.x, 111.x, 112.x, 114.x, 115.x, 116.x, 117.x, 118.x, 320.x, 321.x, 322.x, 324.x, 325.x, 420.x, 421.x, 451.x, 461.x, 462.x, 463.x, 464.x, 465.x, 481.x, 482.x, 485.x, 486.x, 491.21, 494.x, 510.x, 513.x, 540.x, 541.x, 542.x, 562.01, 562.03, 562.11, 562.13, 566.x, 567.x, 569.5x, 569.83, 572.0x, 572.1x, 575.0x, 590.x, 597.x, 599.0x, 601.x, 614.x, 615.x, 616.x, 681.x, 682.x, 683.x, 686.x, 711.0x, 730.x, 790.7x, 996.6x, 998.5x, 999.3x) and ICD-9 codes for acute organ dysfunction, including acute respiratory organ dysfunction (ICD-9 codes 93.90, 96.04, 96.7, 518.81, 518.82, 518.85, 786.09, 799.1), acute cardiovascular organ dysfunction (ICD-9 codes 458.0, 458.8, 458.9, 785.5, 785.51, 785.59, 796.3), acute hematologic organ dysfunction (ICD-9 codes 286.2, 286.6, 286.9, 287.3–287.5, 790.92), acute hepatic organ dysfunction (ICD-9 codes 570, 572.2, 573.3, 573.4), acute kidney injury (ICD-9 codes 39.95, 580.x, 584.x, 586), and acute neurological organ dysfunction (ICD-9 codes 89.14, 293, 348.1, 348.3, 780.01, 780.09) | Definition reported[12, 13] |
| Septic shock | ICD-9 codes 785.5, 785.51, 785.59 plus management codes related to intensive hemodynamic monitoring by central venous catheter insertion (47015B) and administration of inotropic agents (epinephrine, norepinephrine, and dopamine) plus ICD-9 codes used to identify bacterial/fungal infections (001.x, 002.x, 003.x, 004.x, 005.x,008.x, 009.x, 010.x, 011.x, 012.x, 013.x, 014.x, 015.x, 016.x, 017.x, 018.x, 137.x, 020.x, 021.x, 022.x, 023.x, 024.x, 025.x, 026.x, 027.x, 030.x, 031.x, 032.x, 033.x, 034.x, 035.x, 036.x, 037.x, 038.x, 039.x, 040.x, 041.x, 090.x, 091.x, 092.x, 093.x, 094.x, 095.x, 096.x, 097.x, 098.x, 100.x, 101.x, 102.x, 103.x, 104.x, 110.x, 111.x, 112.x, 114.x, 115.x, 116.x, 117.x, 118.x, 320.x, 321.x, 322.x, 324.x, 325.x, 420.x, 421.x, 451.x, 461.x, 462.x, 463.x, 464.x, 465.x, 481.x, 482.x, 485.x, 486.x, 491.21, 494.x, 510.x, 513.x, 540.x, 541.x, 542.x, 562.01, 562.03, 562.11, 562.13, 566.x, 567.x, 569.5x, 569.83, 572.0x, 572.1x, 575.0x, 590.x, 597.x, 599.0x, 601.x, 614.x, 615.x, 616.x, 681.x, 682.x, 683.x, 686.x, 711.0x, 730.x, 790.7x, 996.6x, 998.5x, 999.3x) | Definition reported[14, 15] |
| Pneumonia | Hospitalization for a primary diagnosis of pneumonia alone (ICD-9 codes 480 – 483, 485–487, 507) or a primary diagnosis of respiratory failure (ICD-9 codes 518.8) or sepsis (ICD-9 codes 038) with a secondary diagnosis of pneumonia (ICD-9 codes 480–483, 485–487) | Definition validation[16, 17] |
| Lung abscess | Lung abscess (ICD-9 codes 510, 513) plus procedure codes | Definition validation[18] |
| Infective endocarditis | ICD-9 codes 421.0, 421.1, 421.9 | Definition reported[19] |
| Arteriovenous shunt infection | ICD-9 codes 996.62, 996.63 (may also include necrotizing fasciitis [ICD-9 code 728.86] and cellulitis [ICD-9 code 681 or 682]) | Definition reported[20] |
| Peritoneal dialysis-elated peritonitis | Peritoneal dialysis patients with peritonitis (ICD-9 codes 567,996.68, 999.3) | Definition reported[21] |
| Severe infection-related mortality | 30-day mortality after hospitalization for severe infection events |  |
| Diabetes mellitus | ICD-9 codes 250 | Definition validation[22, 23] |
| Hypertension | ICD-9 codes 401–405 | Definition validation[8, 24] |
| Hyperlipidemia | ICD-9 codes 272 | Definition reported[8, 25] |
| Coronary artery disease | ICD-9 codes 410 – 414 | Definition reported[26] |
| Cerebrovascular disease | ICD-9 codes 430 – 438 | Definition reported[4, 10] |
| Autoimmune disease | ICD-9 codes 710, 714 with catastrophic illness registration | Definition reported[27] |
| Malignancy | ICD-9 codes 140–208 with catastrophic illness registration | Definition reported[2] |
| Alcohol dependence | ICD-9 codes 291, 303, 305.0, 357.5, 425.5, 571.0, 571.1, 571.2, 571.3, 980.0, V11.3 | Definition reported[28] |
| Psychotic disorder | ICD-9 codes 290.8, 290.9, 295, 297–299, 780.1 | Definition reported[29] |
| Anxiety disorder | ICD-9 codes 300.0–300.3 | Definition reported[30] |
| Sleep disorder | ICD-9 codes 307.4, 780.5 | Definition reported[31, 32] |

ICD-9, *International Classification of Disease*, ninth revision.

Reference List

1. Lin TT, Yang YH, Liao MT, Tsai CT, Hwang JJ, Chiang FT, et al. Primary prevention of atrial fibrillation with angiotensin-converting enzyme inhibitors and angiotensin receptor blockers in patients with end-stage renal disease undergoing dialysis. Kidney international. 2015;88(2):378-85. Epub 2015/03/26. doi: 10.1038/ki.2015.96. PubMed PMID: 25807037.

2. Ou SM, Chen YT, Chao PW, Lee YJ, Liu CJ, Yeh CM, et al. Nonsteroidal anti-inflammatory drug use is associated with cancer risk reduction in chronic dialysis patients. Kidney international. 2013;84(1):198-205. Epub 2013/03/15. doi: 10.1038/ki.2013.79. PubMed PMID: 23486520.

3. Li WH, Chen YJ, Tseng WC, Lin MW, Chen TJ, Chu SY, et al. Malignancies after renal transplantation in Taiwan: a nationwide population-based study. Nephrology, dialysis, transplantation : official publication of the European Dialysis and Transplant Association - European Renal Association. 2012;27(2):833-9. Epub 2011/06/03. doi: 10.1093/ndt/gfr277. PubMed PMID: 21633099.

4. Wu CS, Wang SC, Cheng YC, Gau SS. Association of cerebrovascular events with antidepressant use: a case-crossover study. The American journal of psychiatry. 2011;168(5):511-21. Epub 2011/03/17. doi: 10.1176/appi.ajp.2010.10071064. PubMed PMID: 21406464.

5. Cheng CL, Lee CH, Chen PS, Li YH, Lin SJ, Yang YH. Validation of acute myocardial infarction cases in the national health insurance research database in taiwan. Journal of epidemiology / Japan Epidemiological Association. 2014;24(6):500-7. Epub 2014/09/02. PubMed PMID: 25174915; PubMed Central PMCID: PMC4213225.

6. Wu CY, Chan FK, Wu MS, Kuo KN, Wang CB, Tsao CR, et al. Histamine2-receptor antagonists are an alternative to proton pump inhibitor in patients receiving clopidogrel. Gastroenterology. 2010;139(4):1165-71. Epub 2010/07/06. doi: 10.1053/j.gastro.2010.06.067. PubMed PMID: 20600012.

7. Tang CH, Chen TH, Wang CC, Hong CY, Huang KC, Sue YM. Renin-angiotensin system blockade in heart failure patients on long-term haemodialysis in Taiwan. Eur J Heart Fail. 2013;15(10):1194-202. doi: 10.1093/eurjhf/hft082. PubMed PMID: 23671265.

8. Cheng CL, Kao YH, Lin SJ, Lee CH, Lai ML. Validation of the National Health Insurance Research Database with ischemic stroke cases in Taiwan. Pharmacoepidemiology and drug safety. 2011;20(3):236-42. Epub 2011/02/26. doi: 10.1002/pds.2087. PubMed PMID: 21351304.

9. Hsieh CY, Chen CH, Li CY, Lai ML. Validating the diagnosis of acute ischemic stroke in a National Health Insurance claims database. Journal of the Formosan Medical Association = Taiwan yi zhi. 2015;114(3):254-9. Epub 2013/10/22. doi: 10.1016/j.jfma.2013.09.009. PubMed PMID: 24140108.

10. Wu CS, Wang SC, Gau SS, Tsai HJ, Cheng YC. Association of stroke with the receptor-binding profiles of antipsychotics-a case-crossover study. Biological psychiatry. 2013;73(5):414-21. Epub 2012/08/11. doi: 10.1016/j.biopsych.2012.07.006. PubMed PMID: 22877922.

11. Chang CH, Shau WY, Kuo CW, Chen ST, Lai MS. Increased risk of stroke associated with nonsteroidal anti-inflammatory drugs: a nationwide case-crossover study. Stroke; a journal of cerebral circulation. 2010;41(9):1884-90. Epub 2010/07/31. doi: 10.1161/STROKEAHA.110.585828. PubMed PMID: 20671253.

12. Shen HN, Lu CL, Yang HH. Epidemiologic trend of severe sepsis in Taiwan from 1997 through 2006. Chest. 2010;138(2):298-304. Epub 2010/04/07. doi: 10.1378/chest.09-2205. PubMed PMID: 20363844.

13. Angus DC, Linde-Zwirble WT, Lidicker J, Clermont G, Carcillo J, Pinsky MR. Epidemiology of severe sepsis in the United States: analysis of incidence, outcome, and associated costs of care. Critical care medicine. 2001;29(7):1303-10. Epub 2001/07/11. PubMed PMID: 11445675.

14. Chao PW, Shih CJ, Lee YJ, Tseng CM, Kuo SC, Shih YN, et al. Association of postdischarge rehabilitation with mortality in intensive care unit survivors of sepsis. American journal of respiratory and critical care medicine. 2014;190(9):1003-11. Epub 2014/09/12. doi: 10.1164/rccm.201406-1170OC. PubMed PMID: 25210792.

15. Ou SY, Chu H, Chao PW, Ou SM, Lee YJ, Kuo SC, et al. Effect of the use of low and high potency statins and sepsis outcomes. Intensive care medicine. 2014;40(10):1509-17. Epub 2014/08/06. doi: 10.1007/s00134-014-3418-1. PubMed PMID: 25091791.

16. van de Garde EM, Oosterheert JJ, Bonten M, Kaplan RC, Leufkens HG. International classification of diseases codes showed modest sensitivity for detecting community-acquired pneumonia. Journal of clinical epidemiology. 2007;60(8):834-8. Epub 2007/07/04. doi: 10.1016/j.jclinepi.2006.10.018. PubMed PMID: 17606180.

17. Aronsky D, Haug PJ, Lagor C, Dean NC. Accuracy of administrative data for identifying patients with pneumonia. American journal of medical quality : the official journal of the American College of Medical Quality. 2005;20(6):319-28. Epub 2005/11/11. doi: 10.1177/1062860605280358. PubMed PMID: 16280395.

18. Su VY, Liu CJ, Wang HK, Wu LA, Chang SC, Perng DW, et al. Sleep apnea and risk of pneumonia: a nationwide population-based study. CMAJ : Canadian Medical Association journal = journal de l'Association medicale canadienne. 2014;186(6):415-21. Epub 2014/03/05. doi: 10.1503/cmaj.131547. PubMed PMID: 24591276; PubMed Central PMCID: PMC3971026.

19. Shih CJ, Chu H, Chao PW, Lee YJ, Kuo SC, Li SY, et al. Long-term clinical outcome of major adverse cardiac events in survivors of infective endocarditis: a nationwide population-based study. Circulation. 2014;130(19):1684-91. Epub 2014/09/17. doi: 10.1161/CIRCULATIONAHA.114.012717. PubMed PMID: 25223982.

20. Hung YN, Ko PJ, Ng YY, Wu SC. The longevity of arteriovenous graft for hemodialysis patients--externally supported or nonsupported. Clinical journal of the American Society of Nephrology : CJASN. 2010;5(6):1029-35. Epub 2010/04/10. doi: 10.2215/CJN.08181109. PubMed PMID: 20378642; PubMed Central PMCID: PMC2879306.

21. Yang SF, Liu CJ, Yang WC, Chang CF, Yang CY, Li SY, et al. The risk factors and the impact of hernia development on technique survival in peritoneal dialysis patients: a population-based cohort study. Peritoneal dialysis international : journal of the International Society for Peritoneal Dialysis. 2015;35(3):351-9. Epub 2014/03/04. doi: 10.3747/pdi.2013.00139. PubMed PMID: 24584603.

22. Lin CC, Lai MS, Syu CY, Chang SC, Tseng FY. Accuracy of diabetes diagnosis in health insurance claims data in Taiwan. Journal of the Formosan Medical Association = Taiwan yi zhi. 2005;104(3):157-63. Epub 2005/04/09. PubMed PMID: 15818428.

23. Chang CH, Lin JW, Wu LC, Lai MS. Angiotensin receptor blockade and risk of cancer in type 2 diabetes mellitus: a nationwide case-control study. Journal of clinical oncology : official journal of the American Society of Clinical Oncology. 2011;29(22):3001-7. Epub 2011/06/22. doi: 10.1200/JCO.2011.35.1908. PubMed PMID: 21690476.

24. Quan H, Khan N, Hemmelgarn BR, Tu K, Chen G, Campbell N, et al. Validation of a case definition to define hypertension using administrative data. Hypertension. 2009;54(6):1423-8. Epub 2009/10/28. doi: 10.1161/HYPERTENSIONAHA.109.139279. PubMed PMID: 19858407.

25. Tsan YT, Lee CH, Ho WC, Lin MH, Wang JD, Chen PC. Statins and the risk of hepatocellular carcinoma in patients with hepatitis C virus infection. Journal of clinical oncology : official journal of the American Society of Clinical Oncology. 2013;31(12):1514-21. Epub 2013/03/20. doi: 10.1200/JCO.2012.44.6831. PubMed PMID: 23509319.

26. Kuo CF, Yu KH, See LC, Chou IJ, Ko YS, Chang HC, et al. Risk of myocardial infarction among patients with gout: a nationwide population-based study. Rheumatology (Oxford). 2013;52(1):111-7. Epub 2012/07/13. doi: 10.1093/rheumatology/kes169. PubMed PMID: 22787006.

27. Kuo CF, Grainge MJ, Valdes AM, See LC, Luo SF, Yu KH, et al. Familial Aggregation of Systemic Lupus Erythematosus and Coaggregation of Autoimmune Diseases in Affected Families. JAMA internal medicine. 2015. Epub 2015/07/21. doi: 10.1001/jamainternmed.2015.3528. PubMed PMID: 26193127.

28. Lin YT, Wu PH, Lin CY, Lin MY, Chuang HY, Huang JF, et al. Cirrhosis as a risk factor for tuberculosis infection--a nationwide longitudinal study in Taiwan. American journal of epidemiology. 2014;180(1):103-10. Epub 2014/05/16. doi: 10.1093/aje/kwu095. PubMed PMID: 24829509.

29. Lin YT, Wu PH, Kuo MC, Chen CS, Chiu YW, Yang YH, et al. Comparison of dementia risk between end stage renal disease patients with hemodialysis and peritoneal dialysis--a population based study. Scientific reports. 2015;5:8224. Epub 2015/02/24. doi: 10.1038/srep08224. PubMed PMID: 25703589; PubMed Central PMCID: PMC4340159.

30. Lin CH, Lin JW, Liu YC, Chang CH, Wu RM. Risk of Parkinson's disease following anxiety disorders: a nationwide population-based cohort study. European journal of neurology : the official journal of the European Federation of Neurological Societies. 2015. Epub 2015/06/03. doi: 10.1111/ene.12740. PubMed PMID: 26031920.

31. Huang ST, Lin CL, Yu TM, Yang TC, Kao CH. Nonapnea sleep disorders and incident chronic kidney disease: a population-based retrospective cohort study. Medicine. 2015;94(4):e429. Epub 2015/01/31. doi: 10.1097/MD.0000000000000429. PubMed PMID: 25634175.

32. Chung WS, Lin CL, Chen YF, Chiang JY, Sung FC, Chang YJ, et al. Sleep disorders and increased risk of subsequent acute coronary syndrome in individuals without sleep apnea: a nationwide population-based cohort study. Sleep. 2013;36(12):1963-8. Epub 2013/12/03. doi: 10.5665/sleep.3240. PubMed PMID: 24293772; PubMed Central PMCID: PMC3825447.
