# Supplementary material for: Depression amongst patients commencing maintenance dialysis is associated with increased risk of death and severe infections: A nationwide cohort study
Source: PLoS One. 2019 Jun 13;14(6):e0218335. doi: 10.1371/journal.pone.0218335 (PMC6564035; doi:10.1371/journal.pone.0218335)
Supplement: S2 Table — (DOCX) [file pone.0218335.s003.docx]

**S2 Table. Anatomical Therapeutic Chemical (ATC) code used to define medication use**

| Drug type | ATC classification system codes | Drug name |
| --- | --- | --- |
| Antidepressants | N06A | Fluoxetine, Citalopram, Paroxetine, Sertraline, Fluvoxamine, Escitalopram, Imipramine, Clomipramine, Amitriptyline, Doxepin, Dosulepin, Maprotiline, Melitracen, Nortriptyline, Moclobemide, Duloxetine, Milnacipran, Venlafaxine, Trazodone, Mirtazapine, Bupropion |
| Antiplatelets/warfarin | B01AC04, B01AC05, B01AC06, B01AA03 | Clopidogrel, Ticlopidine, Acetylsalicylic acid (Aspirin), Warfarin |
| Antihypertension drugs (angiotensin-converting enzyme inhibitors/angiotensin receptor blockers, beta-blockers, calcium channel blockers, alpha-blockers) | C09A, C09B, C09C, C09D, C07A, C08C, C08D, C08E, C02CA, C02AC01, C02CC02, C02AB | Captopril, Enalapril, Lisinopril, Perindopril, Ramipril, Quinapril, Benazepril, Cilazapril, Fosinopril, Imidapril, Candesartan, Irbesartan, Losartan, Olmesartan, Telmisartan, Valsartan, Labetalol, Pindolol, Acebutolol, Alprenolol, Atenolol, Betaxolol, Bisoprolol, Carteolol, Carvedilol, Nadolol, Metoprolol, Oxprenolol, Propranolol, Sotalol, Timolol, Metipranolol, Esmolol, Nifedipine, Nicardipine, Felodipine, Amlopidine, Isradipine, Diltiazem, Verapamil, Doxazosin, Prazosin, Terazosin, Bunazosin, Clonidine, Guanethidine, Methyldopa |
| Statins | C10AA | Atorvastatin, Fluvastatin, Lovastatin, Pravastatin, Rosuvastatin, Simvastatin |
| Oral antidiabetic agents | A10B | Acarbose, Acetohexamide, Buformin, Chlorpropamide, Gliclazide, Glimepiride, Glipizide, Gliquidone, Glyburide, Metformin, Nateglinide, Pioglitazone, Repaglinide, Rosiglitazone, Tolazamide, Tolbutamide, Sitagliptin, Miglitol |
| Insulin | A10A | Insulin human, Insulin zinc crystal, Insulin chromatography, Insulin monocomponent, Insulin isophane, Insulin protamine, Insulin lispro, Insulin glargine, Insulin aspart, Insulin glulisine, Insulin detemir |
| Antipsychotic agents | N05A | chlorpromazine, clopenthixol, clothiapine, flupenthixol, fluphenazine, haloperidol, levomepromazine, loxapine, methotrimeprazine, perphenazine, pimozide, pipotiazine, prochlorperazine, sulpiride, trifluoperazine, thioridazine, amisulpride, aripiprazole, clozapine, olanzapine, quetiapine, risperidone, ziprasidone, and zotepine |
| Benzodiazepines | N05B | Diazepam, Chlordiazepoxide, Medazepam, Oxazepam, Clorazepate, Lorazepam, Bromazepam, Clobazam, Alprazolam, Nordazepam, Fludiazepam |
| Hypnotics | N05C | Secobarbital, Flurazepam, Nitrazepam, Flunitrazepam, Estazolam, Triazolam, Midazolam, Brotizolam, Zopiclone, Zolpidem, Zaleplon, Dexmedetomidine |
