# Supplementary material for: Depression amongst patients commencing maintenance dialysis is associated with increased risk of death and severe infections: A nationwide cohort study
Source: PLoS One. 2019 Jun 13;14(6):e0218335. doi: 10.1371/journal.pone.0218335 (PMC6564035; doi:10.1371/journal.pone.0218335)
Supplement: S3 Table — (DOCX) [file pone.0218335.s004.docx]

**S3 Table. Incidence and incidence rate ratio of study outcomes among incident dialysis patients**

| **Variable** | **Depression** | | | **Control** | | |  |
| --- | --- | --- | --- | --- | --- | --- | --- |
|  | **N** | **PY** | **I** | **N** | **PY** | **I** | **Incidence rate ratio (95% CI)** |
| **All-cause mortality** | 1,121 | 7,882 | 142.22 | 4,890 | 43,943 | 111.28 | 1.28 (1.20 - 1.36)*** |
| **Major cardiovascular events** | 806 | 8,568 | 94.07 | 3,787 | 46,334 | 81.73 | 1.15 (1.07 - 1.24)*** |
| Acute coronary syndrome | 234 | 10,627 | 22.02 | 1,103 | 56,214 | 19.62 | 1.12 (0.97 - 1.29) |
| Heart failure | 489 | 9,704 | 50.39 | 2,270 | 51,593 | 44 | 1.15 (1.04 - 1.26)** |
| Ischemic stroke | 225 | 10,599 | 21.23 | 1,031 | 56,002 | 18.41 | 1.15 (1.00 - 1.33) |
| Hemorrhagic stroke | 91 | 11,040 | 8.24 | 434 | 58,378 | 7.43 | 1.11 (0.88 - 1.39) |
| **Fatal infections** | 513 | 9,853 | 52.06 | 2,100 | 53,295 | 39.4 | 1.32 (1.20 - 1.46)*** |
| **Severe infections** | 1,140 | 7,497 | 152.06 | 4,990 | 42,636 | 117.04 | 1.30 (1.22 - 1.39)*** |
| Sepsis | 592 | 9,500 | 62.31 | 2,408 | 52,298 | 46.04 | 1.35 (1.24 - 1.48)*** |
| Septic shock | 183 | 10,819 | 16.91 | 637 | 57,863 | 11.01 | 1.54 (1.30 - 1.81)*** |
| Pneumonia | 465 | 9,872 | 47.1 | 1,804 | 53,823 | 33.52 | 1.41 (1.27 - 1.56)*** |
| Lung abscess | 16 | 11,301 | 1.42 | 78 | 59,481 | 1.31 | 1.08 (0.63 - 1.85) |
| Infective endocarditis | 25 | 11,255 | 2.22 | 104 | 59,345 | 1.75 | 1.27 (0.82 - 1.96) |
| Arteriovenous shunt infection | 476 | 9,804 | 48.55 | 2,105 | 52,361 | 40.2 | 1.21 (1.09 - 1.33)*** |
| Peritoneal dialysis-related peritonitis | 149 | 10,870 | 13.71 | 704 | 57,456 | 12.25 | 1.12 (0.94 - 1.34) |

PY, person-years; I, incidence.

Incident rate (per 1000 person-years).
