# Supplementary material for: Depression amongst patients commencing maintenance dialysis is associated with increased risk of death and severe infections: A nationwide cohort study
Source: PLoS One. 2019 Jun 13;14(6):e0218335. doi: 10.1371/journal.pone.0218335 (PMC6564035; doi:10.1371/journal.pone.0218335)
Supplement: S4 Table — (DOCX) [file pone.0218335.s005.docx]

**S4 Table. Risks of single cardiovascular events associated to depression**

| **Variable** | **Hazard Ratio (95% CI)** | |
| --- | --- | --- |
|  | **Model 1^†^** | **Model 2^‡^** |
| Acute coronary syndrome | 1.02 (0.88 - 1.18) | 1.05 (0.90 - 1.22) |
| Heart failure | 1.04 (0.94 - 1.15) | 1.02 (0.92 - 1.14) |
| Ischemic stroke | 1.00 (0.86 - 1.16) | 0.98 (0.84 - 1.14) |
| Hemorrhagic stroke | 1.08 (0.85 - 1.37) | 1.09 (0.86 - 1.39) |

^†^Model 1: Adjusted for comorbid disorders (diabetes mellitus, hypertension, hyperlipidemia, coronary artery disease, cerebrovascular disease, autoimmune disease, malignancy, alcohol dependence, psychotic disorder, anxiety disorder, sleep disorder), and competing risk of mortality

^‡^Model 2: Adjusted for comorbid disorders, medications (antiplatelets/warfarin, anti-hypertensive drugs, statins, oral antidiabetic agents, insulin, antipsychotic agents, benzodiazepines, hypnotics), and competing risk of mortality

^§^Hazard Ratio of cardiovascular and infection events was used subdistribution hazard model.

*p<0.05, **p<0.01, ***p<0.001
