# Supplementary material for: Depression amongst patients commencing maintenance dialysis is associated with increased risk of death and severe infections: A nationwide cohort study
Source: PLoS One. 2019 Jun 13;14(6):e0218335. doi: 10.1371/journal.pone.0218335 (PMC6564035; doi:10.1371/journal.pone.0218335)
Supplement: S5 Table — (DOCX) [file pone.0218335.s006.docx]

**S5 Table. Sensitivity analyses using alternative algorithms to identify cases with depression**

| **Analyses** | **Hazard Ratio (95% CI)^†^** | | | | | |
| --- | --- | --- | --- | --- | --- | --- |
|  | All-cause mortality | Fatal infection | Severe infection | Sepsis | Septic shock | Pneumonia |
| **Main analysis** | 1.24 (1.16 - 1.33)*** | 1.22 (1.10 - 1.36)*** | 1.13 (1.06 - 1.22)*** | 1.19 (1.08 - 1.31)*** | 1.36 (1.13 - 1.62)*** | 1.20 (1.07 - 1.34)** |
| **Approach 1** | 1.34 (1.20 - 1.50)*** | 1.24 (1.04 - 1.49)* | 1.17 (1.04 - 1.31)** | 1.16 (0.98 - 1.36) | 1.44 (1.06 - 1.94)* | 1.18 (0.97 - 1.42) |
| **Approach 2** | 1.22 (1.13 - 1.32)*** | 1.22 (1.08 - 1.37)*** | 1.14 (1.05 - 1.23)*** | 1.15 (1.03 - 1.29)* | 1.34 (1.09 - 1.64)** | 1.19 (1.05 - 1.35)** |
| **Approach 3** | 1.24 (1.12 - 1.36)*** | 1.27 (1.10 - 1.48)*** | 1.16 (1.05 - 1.27)** | 1.24 (1.09 - 1.42)** | 1.42 (1.11 - 1.81)** | 1.25 (1.07 - 1.46)** |
| **Approach 4** | 1.26 (1.16 - 1.38)*** | 1.22 (1.06 - 1.39)** | 1.12 (1.03 - 1.22)* | 1.17 (1.04 - 1.33)* | 1.22 (0.97 - 1.54) | 1.21 (1.05 - 1.39)** |

^†^Adjusted for comorbidities (diabetes mellitus, hypertension, hyperlipidemia, coronary artery disease, cerebrovascular disease, autoimmune disease, malignancy, alcohol dependence, psychotic disorder, anxiety disorder, sleep disorder), medications (antiplatelets/warfarin, anti-hypertensive drugs, statins, oral antidiabetic agents, insulin, antipsychotic agents, benzodiazepines, hypnotics), and competing mortality (when appropruate).

Approach 1: redefining depression diagnosis as the major depression diagnostic codes (ICD-9 codes 296.2 and 296.3)

Approach 2: redefining depression diagnosis as the presence of any depression diagnostic code in at least two outpatient claims or one inpatient claim, the use of any depression-related medication

Approach 3: redefining depression diagnosis as the presence of any depression diagnostic code in at least two outpatient claims or one inpatient claim and psychiatrists outpatient visits

Approach 4: propensity score-matched approach

^§^All-cause mortality was used Cox-proportional hazard model; cardiovascular or infection events were used subdistribution hazard model

*p<0.05, **p<0.01, ***p<0.001
