# Supplementary figures and images for: Depression amongst patients commencing maintenance dialysis is associated with increased risk of death and severe infections: A nationwide cohort study
Source: PLoS One. 2019 Jun 13;14(6):e0218335. doi: 10.1371/journal.pone.0218335 (PMC6564035; doi:10.1371/journal.pone.0218335)

**S1 Fig. Stratified analysis for death associated with depression**


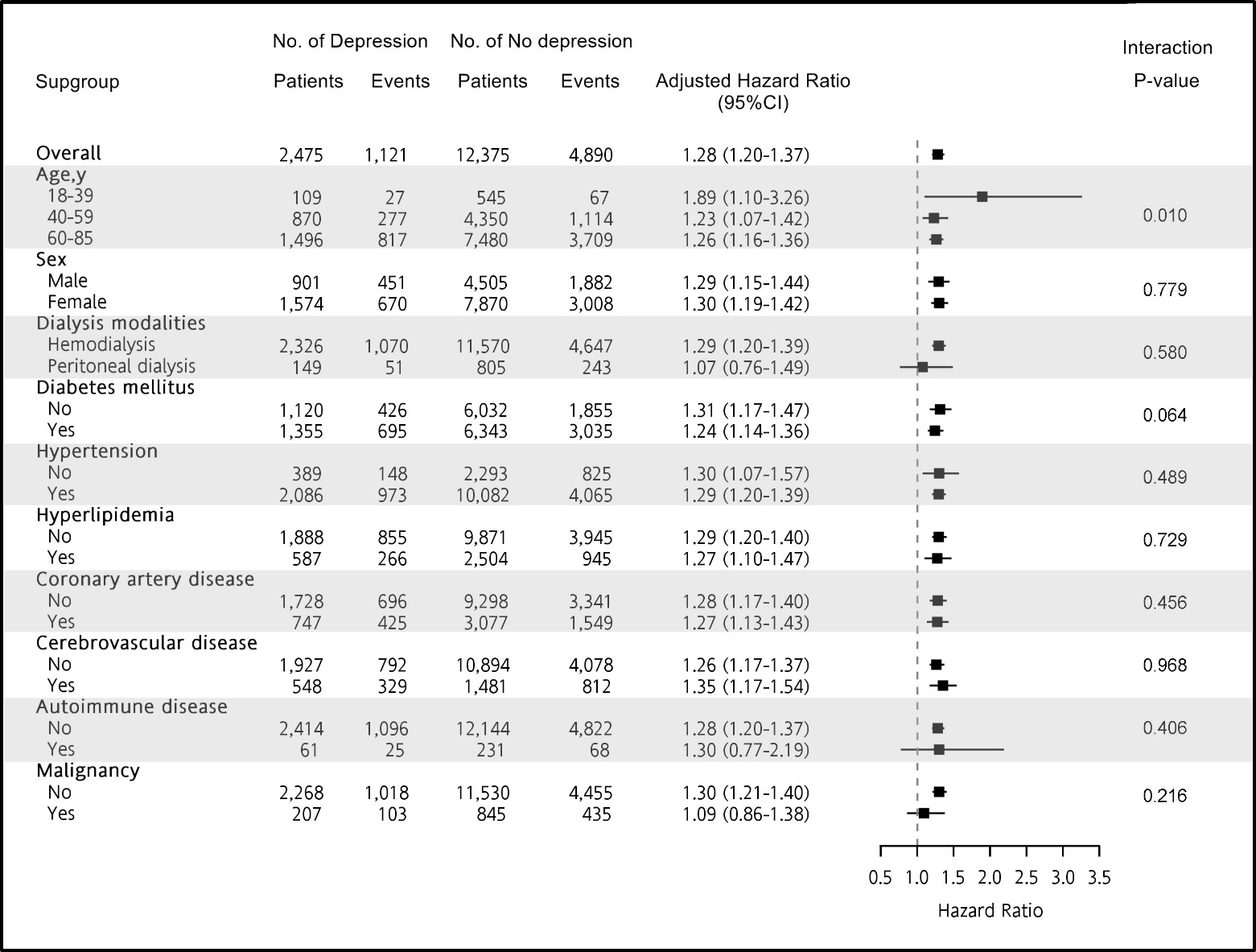

Supplement: S1 Fig — (DOCX) [file pone.0218335.s007.docx]

**S2 Fig. Stratified analysis for major cardiovascular events associated with depression**


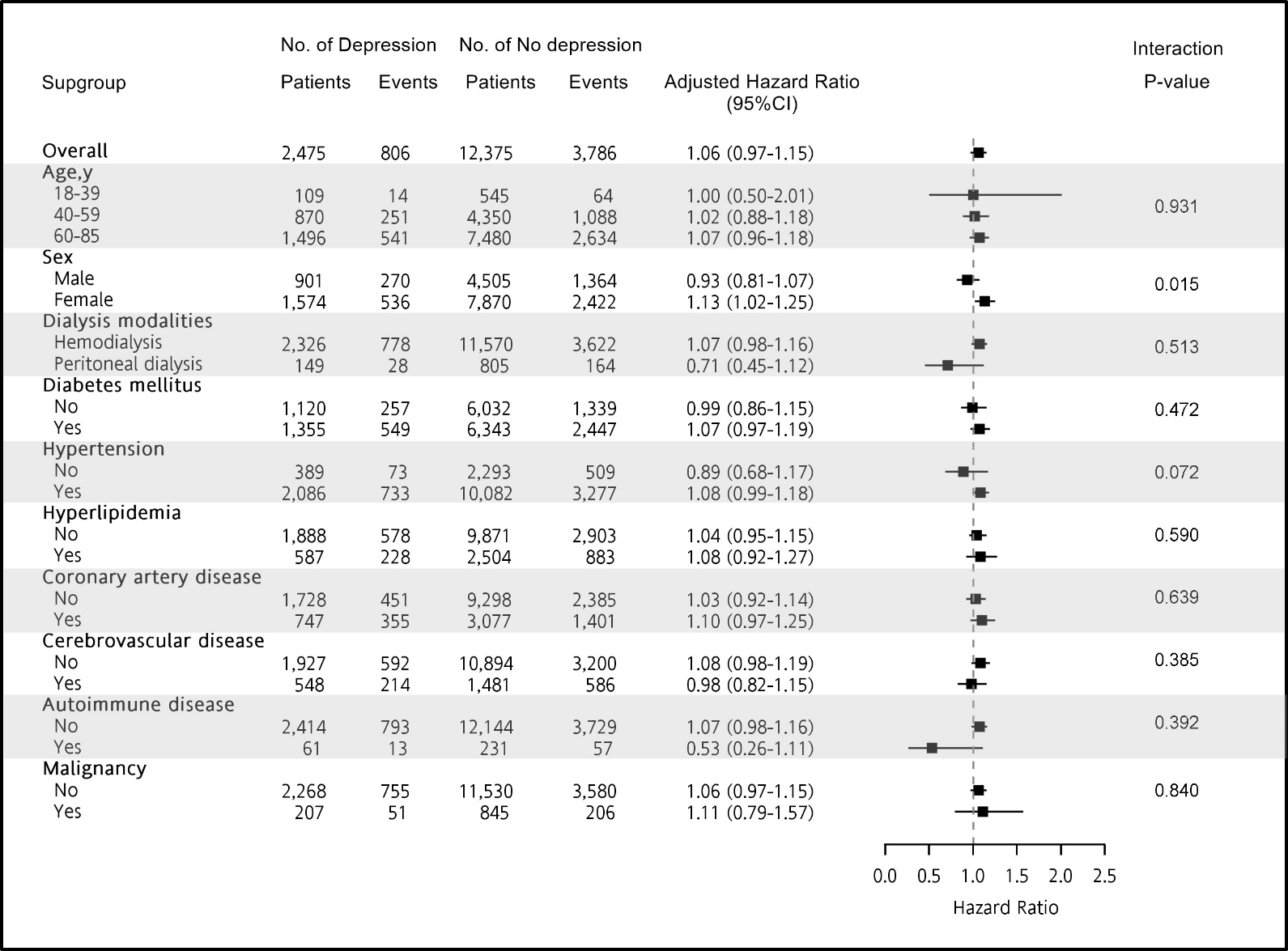

Supplement: S2 Fig — (DOCX) [file pone.0218335.s008.docx]

**S3 Fig. Stratified analysis for severe infections associated with depression**


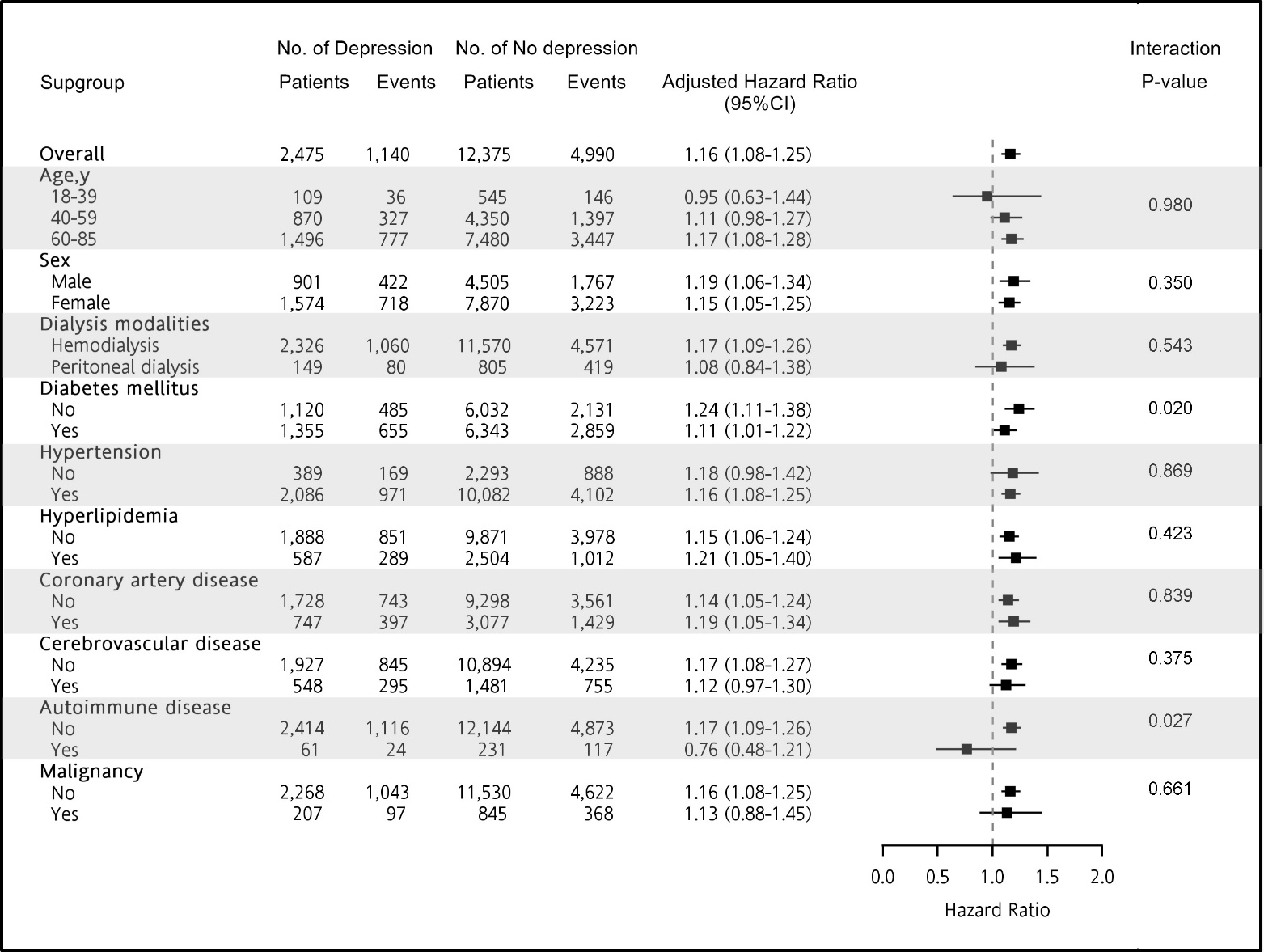

Supplement: S3 Fig — (DOCX) [file pone.0218335.s009.docx]

**S4 Fig. Stratified analysis for fatal infections associated with depression**


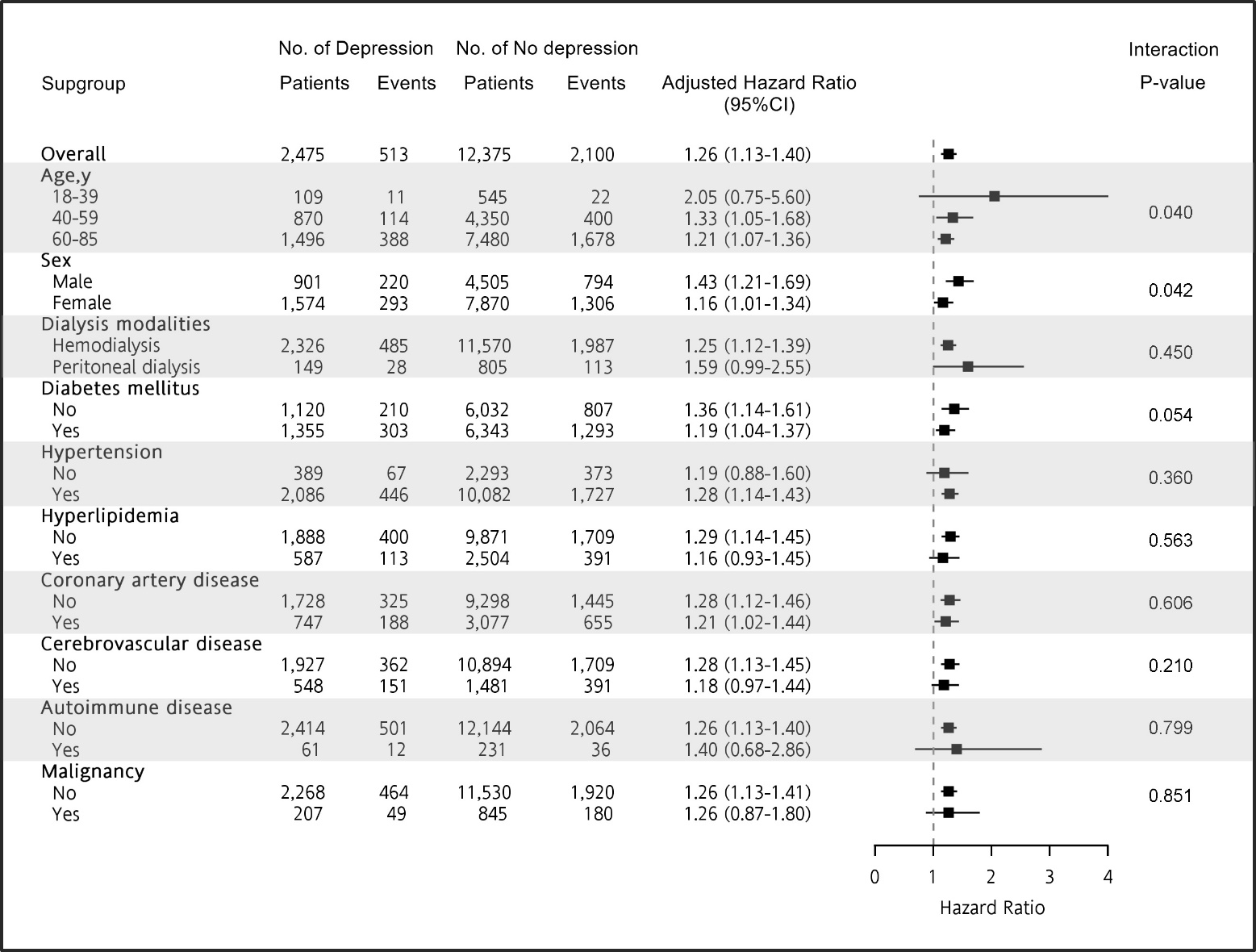

Supplement: S4 Fig — (DOCX) [file pone.0218335.s010.docx]
